# Understanding Health Professionals’ Migration in Bulgaria: Driving and Retention Factors Among Physicians and Nurses †

**DOI:** 10.3390/healthcare13212723

**Published:** 2025-10-28

**Authors:** Iliyana Linkova, Maria Rohova

**Affiliations:** Department of Healthcare Economics and Management, Medical University of Varna, 9002 Varna, Bulgaria; mariarohova@abv.bg

**Keywords:** health professionals, migration, driving factors, retention determinants, Eastern Europe, brain drain

## Abstract

**Background**: In recent decades, the emigration of Bulgarian health professionals has become a persistent challenge driven by multiple interplaying factors. A comprehensive understanding of these key determinants is essential for the development of targeted retention strategies. This study examines the primary factors influencing the migration and retention of physicians and nurses in Bulgaria. **Methods**: A cross-sectional study was conducted in 2022 involving 338 health professionals without professional experience abroad. Data were collected using an online questionnaire administered by a polling agency. To identify the underlying structure of the driving and retention factors, we conducted Exploratory Factor Analysis (EFA). Subsequently, the Mann–Whitney U test was employed to examine the differences in retention and motivational factors between physicians and nurses. **Results**: EFA of the migration drivers identified a three-factor structure, explaining 70.34% of the total variance: professional advancement and work environment; financial incentives; and family benefits. The analysis of retention determinants revealed three distinct constructs, explaining 54.46% of the variance: socioeconomic and healthcare framework; employment and career development; and personal considerations and social environment. The Mann–Whitney U test indicated that the impact of financial incentives on migration decisions exerted a weaker influence on physicians’ intentions (*r* = −0.23). Personal considerations and the social environment emerged as more significant retention determinants for nursing professionals (*r* = −0.15). **Conclusions**: This study extends current understanding by identifying the underlying constructs of driving and retention factors in health professional migration. The findings underscore the need for evidence-based interventions to mitigate brain drain and retain skilled professionals.

## 1. Introduction

Migration of health professionals is a multifaceted phenomenon that reflects the existing imbalances in the workforce market [1]. Factors related to supply and demand dynamics within the health sector either encourage or discourage migration [2,3,4]. On the supply side, economic conditions, such as wage disparities and living standards, frequently motivate professionals to seek better opportunities abroad [5,6,7]. Professional development opportunities, advanced training, and career progression pathways in destination countries further influence migration decisions [8]. On the demand side, acute shortages in certain areas and medical specialties create imbalances in health services provision, prompting international recruitment to fill these gaps. In the United Kingdom, for instance, the National Health Service depends on foreign physicians, including those originating from Southern and Eastern European countries, such as Italy, Greece, and Romania [9], which themselves face significant shortages. These challenges are exacerbated by the aging health workforce, as a substantial proportion of professionals approaches retirement age, thereby intensifying the need for skilled replacements.

A well-functioning national health system shapes professionals’ migration choices by influencing job satisfaction, career growth, and working conditions [8]. These factors directly impact the retention of skilled health workers, contributing to sustained workforce motivation and commitment. Furthermore, licensing, training, and workforce planning policies shape migration decisions. Collectively, these determinants contribute to the ongoing challenge of retaining skilled health professionals and influence the distribution of medical expertise across regions.

Research conducted between 2017 and 2023 demonstrates that health professional migration within the European Union (EU) simultaneously increases destination countries’ dependence on foreign-trained workers and exacerbates workforce shortages in source countries. The main countries of origin are Romania, Slovakia, Spain, Lithuania, Latvia, Portugal, Bulgaria, Greece, Hungary, Italy, and Slovenia [10]. Bulgaria, as a significant source of health workforce, serves as a pertinent example of these dynamics, particularly in the context of its accession to the EU. The removal of labor mobility restrictions facilitates migration to Western European countries [11,12]. This trend is primarily driven by disparities in health financing, which, despite improvements, remains significantly lower than in other EU member states. Underinvestment in infrastructure, outdated medical equipment, and inadequate remuneration contribute to dissatisfaction among health professionals, prompting them to seek better working conditions and higher wages abroad [13]. Furthermore, limited prospects for professional advancement incentivize outflows to member states where health workers can achieve superior career growth and development. Migration extends beyond intra-EU mobility, with practitioners from non-EU Balkan countries migrating to EU member states despite qualification recognition challenges [14].

According to the World Health Organization (WHO), a substantial part of the physician and nurse migration flows within the European Region originates from the Southern and Eastern European subregions [15]. These trends have profound implications for the health systems. Over the past decade, the Bulgarian health system has been facing a severe shortage of nursing staff [12], a crisis that threatens the system’s resilience and sustainability while hindering access to essential health services. Although Bulgaria has a relatively high physician density compared to other EU countries, this apparent advantage is offset by disparities in distribution across regions and specialties [16]. Organization for Economic Co-operation and Development (OECD) data on health workers’ migration reveals significant physician outflows from Bulgaria to member countries, with a pronounced surge during the COVID-19 pandemic [17]. The pandemic has intensified this trend, as many OECD countries have faced acute shortages of health workers and implemented policies to actively recruit professionals from abroad [18]. This migration pattern has exacerbated existing staff shortages, placing additional strain on the remaining workforce and diminishing the overall efficiency of health services. To address workforce shortages, the WHO recommends implementing retention-focused strategies for health workers. Romania offers a pertinent example, with targeted interventions reducing physician emigration and increasing workforce density over the past decade [15].

The increased emigration of physicians and nurses from Bulgaria and other Southern and Eastern European countries can be attributed to a variety of factors, underscoring the need for targeted policies to tackle the root causes and develop strategies to attract and retain health professionals. This study examined the primary factors influencing the migration and retention of physicians and nurses in Bulgaria and the differences in these factors between the two professional groups. Addressing driving and enhancing retention factors can significantly mitigate brain drain and contribute to a more stable and resilient health system, benefiting professionals and the population.

Drawing on quantitative evidence from the Bulgarian case study within the EU-funded Mobility of Health Professionals (MOHPROF) project (2008–2012) [19], two hypotheses were formulated. First, migration incentives, particularly financial motivations, are expected to differ significantly between physicians and nurses. Second, significant differences in retention motives between physicians and nurses are expected, driven primarily by family and community ties.

## 2. Materials and Methods

### 2.1. Study Design and Sample Selection

The present study employed a cross-sectional survey design to examine the factors influencing the retention of health professionals in Bulgaria and their intention to work abroad. The survey was conducted between May and June 2022 by a polling agency.

The sample was selected using stratified random sampling to ensure that the distribution of respondents accurately reflected the population of health workers in Bulgaria. Two criteria were used to design the sample: profession (physicians, dentists, nurses, and midwives) and place of residence (capital, district city, small town, and village). Categorizations by profession facilitated an understanding of how factors might vary across professional groups. The distinction by geographical location acknowledged the potential influence of regional disparities on career decisions and retention. The population for the study consisted of physicians, dentists, and nursing professionals (nurses and midwives): approximately 29,000 physicians, 7500 dentists, and 32,100 nurses and midwives in 2021 [20]. The final sample consisted of 447 health professionals, providing a robust dataset for the analysis. Among the 447 respondents, two distinct cohorts emerged: health workers who had been abroad and those without such experiences. The present study focused on the latter cohort (n = 338), as the motivation for migration was expected to differ between the two groups.

An a priori power analysis determined minimum sample size requirements using G*Power (v3.1). Given the limited existing research on health workforce migration in Bulgaria, conservative assumptions were applied, consistent with social science research conventions: medium effect size (Cohen’s d = 0.5) [21], statistical power of 0.80, and significance level of α = 0.05 for independent samples comparison. This yielded a minimum required sample of 92 participants per professional group. The achieved sample of 338 health workers (physicians and dentists: n = 191; nurses and midwives: n = 147) exceeded this requirement, ensuring sufficient statistical power for between-group comparisons of migration determinants and retention factors.

### 2.2. Data Collection

Data were gathered through an online self-administered questionnaire disseminated to the participants via email and professional networks. The online format was chosen for convenience and efficiency while ensuring anonymity and confidentiality.

The questionnaire was developed based on migration theories, specifically drawing from Lee’s push-pull model. This model analyzes migration motives by differentiating between “push” factors in the countries of origin and “pull” factors in the countries of destination [22]. Furthermore, a multi-level framework was adopted, categorizing influencing factors into three levels: the macro-level, encompassing country-wide considerations and the national health system; the meso-level, including profession-specific factors; and the micro-level, addressing individual considerations [23]. Health worker migration is driven by relatively stable macro- and meso-level factors. While financial incentives and career opportunities have remained consistently dominant drivers, research from the early 2000s onward demonstrates that quality of life, working conditions, and security concerns have emerged as crucial determinants of migration decisions [24].

Instrument design was informed by findings from the Bulgarian component of the EU-funded MOHPROF project, which investigated mobility trends among physicians and nurses through data analyses and semi-structured interviews. These interviews explored profession-specific migration drivers and retention factors [8]. The current survey instrument operationalizes migration determinants identified in the interviews with Bulgarian health professionals [25], structured according to the multi-level analytical framework to enable quantitative measurement [23]. Following the initial development of driving and retention factor sets, consultations were conducted with MOHPROF’s Bulgarian expert team to assess the relevance and appropriateness of the included variables. Based on the recommendations, the final set of motivational and retention factors was refined to ensure content validity of the instrument.

The questionnaire comprised two primary sections:

(1) Intentions to work abroad (driving factors)—this section included 12 items related to the factors influencing migration intentions. Each item was rated by participants on a four-point scale ranging from “no influence” (1) to “very strong influence” (4).
(2) Retention factors—the second section included 14 factors shaping health professionals’ decisions to remain in their current positions. The participants rated each item on the same four-point scale.

### 2.3. Data Analysis

Data analysis was conducted using several statistical methods and proceeded in the following steps:

(1) Reliability testing

A preliminary reliability test was conducted using an ordinal version of Cronbach’s alpha for Likert-type scales. A Cronbach’s alpha value of 0.70 or higher was considered acceptable for confirming the reliability of the scales.

Based on the reliability test, one item from the retention factors was removed, leaving a total of 13 items. This refinement enhanced the overall internal consistency and resulted in an increase in the alpha value to 0.93. No significant improvement in the overall reliability (α = 0.95) was observed after removing any item from the factors influencing migration intentions, and the final set included 12 items.

(2) Exploratory Factor Analysis

To identify underlying constructs within the retention factors and intentions to work abroad, we conducted Exploratory Factor Analysis (EFA). EFA utilized Principal Axis Factoring, as this extraction method is particularly appropriate when inter-item correlations are expected.

The suitability of the dataset for EFA was first evaluated using the Kaiser-Meyer-Olkin (KMO) measure and Bartlett’s test of sphericity. A KMO value above 0.6 and a significant Bartlett’s test (*p* < 0.05) confirmed the appropriateness of the data for factor analysis. Factor retention was then guided by eigenvalues, scree plot, and parallel analysis results. Promax rotation was employed to permit correlations between the factors, thereby facilitating a clearer interpretation of the factor structure. A cut-off value of 0.4 was applied to determine the appropriate item loadings [26]. Items exhibiting low item-total correlations (below 0.4) were removed, thereby improving the overall internal reliability of the scales.

(3) Variable extraction and statistical testing

Following the EFA and reliability assessments, migration drivers and retention factors were extracted as composite variables for further analysis.

Health professionals were stratified into two groups: the first comprising physicians and dentists, and the second encompassing nurses and midwives. This grouping allowed for a comparative analysis of the identified factors across distinct professional categories. The extracted variables, stratified by professional category, were assessed for normality using the Shapiro–Wilk test. As the assumption of normality was not met, the Mann–Whitney U test was applied to evaluate the differences in retention and driving factors between professional groups. A significance level of *p* < 0.05 was set, and effect sizes were subsequently calculated for the statistically significant findings.

Statistical analyses were performed using SPSS, version 26.

### 2.4. Ethics Statement

Ethical approval was granted by the Research Ethics Committee of Medical University–Varna (Ref. No. 115 from 31 March 2022). Participants were provided with information about the study aim, the voluntary nature of their involvement, and procedures ensuring anonymity and data confidentiality. All participants provided electronic informed consent before accessing the online questionnaire.

## 3. Results

### 3.1. Demographic Characteristics of the Respondents

Demographic characteristics of the sample are presented in Table 1. A majority of the participants were physicians (46.8%) with over 20 years of professional experience (51.8%), predominantly within the 46–55 age group (32.8%). Female participants predominated (78.4% vs. 21.6%), consistent with health sector feminization trends. Nearly half of the participants (49.4%) resided in district cities, while 28.7% were based in the capital. Most were married (56.5%) and had children.

### 3.2. Factors Influencing Migration Intentions (Drivers)

EFA was conducted using 12 variables to examine the latent dimensions underlying migration intentions among health professionals in Bulgaria. Sampling adequacy was assessed using the Kaiser–Meyer–Olkin test, yielding a value of 0.884. Bartlett’s test of sphericity demonstrated a significant correlation between the variables (χ^2^ (66) = 3234.51; *p* < 0.001). The number of factors to extract was determined using multiple methods. Parallel analysis indicated four factors, which served as the maximum threshold for exploratory factor extraction. However, both Kaiser’s criteria [27] and the scree plot method [28] suggested the extraction of three factors, collectively accounting for 70.34% of the total variance. The fourth factor identified by parallel analysis had an eigenvalue of less than 1.0, and its inclusion did not significantly increase the cumulative variance. Therefore, we retained three factors representing distinct dimensions of health professionals’ migration intentions (Table 2).

The first factor, labeled “Professional advancement and work environment”, exhibited a strong correlation with six items, with access to advanced technologies demonstrating the highest loading (0.930). Two variables were grouped under the second factor, “Financial incentives”. The third factor, “Family benefits”, was associated with two items reflecting family-related considerations in migration decisions. Except for one item, the retained variables displayed robust loadings on the respective factors (>0.60). This pattern indicates a strong item-factor correlation, supporting the construct validity of the extracted factor structure. The item “Financial support for the family (remittances)” failed to meet the minimum factor-loading threshold (0.40) and was excluded from the final model.

Financial incentives (Factor 2) and family benefits (Factor 3) were less motivating, explaining 8.96% and 7.43% of the total variance, respectively.

Following the extraction of the factors, a reliability test was conducted. The ordinal alpha coefficients were high (Factor 1: α = 0.95; Factor 2: α = 0.97; Factor 3: α = 0.94), indicating excellent internal consistency.

### 3.3. Retention Factors

EFA was performed on the retention factors, encompassing 13 variables. The Kaiser-Meyer-Olkin measure (KMO = 0.909) and Bartlett’s test of sphericity (χ^2^ (78) = 2389.80; *p* < 0.001) demonstrated the suitability of the data for factor analysis. Parallel analysis suggested the retention of four factors, whereas both Kaiser’s criterion and the scree plot method supported the extraction of two factors. Nevertheless, the third factor had an eigenvalue of 0.949, leading to its retention. Consequently, the model’s explanatory power increased to 54.46%, offering a more comprehensive understanding of the underlying structure.

The first factor, accounting for 45.91% of the cumulative variance, exhibited a strong association with five items pertaining to the socioeconomic context and health system organization (Table 3). These variables constitute key incentives for health professionals to stay in Bulgaria, underscoring the substantial influence of this factor on workforce retention. The second factor, comprising four items, collectively explained 4.33% of the total variance, thus highlighting the importance of workplace-related considerations in retention decisions. The third factor was correlated with three variables, focusing on safety, social connections, and communication barriers as critical determinants of health professionals’ decisions. This factor accounted for 4.22% of the total variance. However, the item “Local schooling opportunities” demonstrated cross-loadings with both Factor 1 and Factor 3, resulting in its exclusion from the final model.

Cronbach’s alpha values for Factor 1 (α = 0.89) and Factor 2 (α = 0.92) demonstrated high internal consistency. Factor 3 exhibited acceptable reliability (α = 0.72), meeting the minimum criterion for internal consistency.

### 3.4. Comparison Between Physicians and Nurses

Differences in migration intentions between physicians (including dentists) and nursing professionals were examined using Mann–Whitney U tests for each of the three factor scores. No statistically significant differences emerged for Factor 1 “Professional advancement and work environment” (U = 12,859, *p* = 0.184) or Factor 3 “Family benefits” (U = 12,577, *p* = 0.100), indicating that professional opportunities, health system advantages, and family benefits exerted similar influences on migration intentions across both groups. A significant difference was observed for Factor 2 “Financial incentives” (U = 10,221, *p* < 0.001), yielding a small-to-medium effect (rank biserial *r* = −0.23). Thus, financial incentives emerged as a more salient migration determinant for nursing professionals compared to physicians.

Mann–Whitney U tests revealed no significant differences between physicians and nursing professionals in retention motives associated with Factor 1 “Socioeconomic and healthcare framework” (U = 12,812; *p* = 0.168), or Factor 2 “Employment and career development” (U = 14,014; *p* = 0.978). A significant difference was observed for Factor 3 “Personal considerations and social environment” (U = 11,586, *p* = 0.006, *r* = −0.15). These findings suggest that physicians attributed relatively less importance to aspects such as security, language barriers, or opportunities for familial proximity compared to nursing professionals.

## 4. Discussion

Our study identified three principal factors influencing health professionals’ migration decisions: (1) the health system and professional opportunities in the destination country; (2) financial incentives; and (3) family-related benefits of working abroad. These findings align with recent research in Eastern Europe [7,29,30,31,32,33,34], which demonstrate that migration decisions are shaped by multiple factors—including economic considerations, professional opportunities, family and social networks, and systemic and policy factors.

In addition to migration drivers, our study explored the retention factors in Bulgaria. The identified latent constructs included (1) factors related to socioeconomic and healthcare frameworks; (2) employment and career development; and (3) personal considerations and social environment. Notably, these findings are consistent with prior research highlighting key incentives and effective strategies for retaining health professionals [31,32,33,35,36,37].

The economic determinants of migration from Eastern to Western Europe are complex and deeply embedded in the disparities between these regions. The pronounced income gap between countries provides a strong incentive for professionals to seek employment abroad. While the importance of economic factors may vary across national contexts, they consistently emerge as a critical driver in the broader landscape of East–West European migration patterns [7,33,34,38]. According to a study conducted in Poland, financial considerations are identified as the predominant factor influencing nursing students’ migration intentions [30]. Our findings corroborate Polish evidence while revealing professional stratification: financial incentives exhibited more substantial influence among nurses compared to physicians. Thus, competitive and satisfactory salaries can be considered a significant retention factor in Bulgaria and other Central and Eastern European countries facing similar workforce challenges.

Furthermore, the perceived instability of the labor market reinforces migration motivation [30]. The prospect of more stable employment opportunities combined with superior working conditions is a compelling pull factor for skilled workers.

Favorable working conditions abroad emerged as key migration drivers in our analysis, interacting with other determinants of the working environment. These findings align with previous studies within the Romanian and Polish contexts, highlighting poor working conditions as an important push factor for health worker migration [29,30]. Unfavorable conditions include high workload, frequent workplace conflicts, and inadequate leadership [33,39]. Therefore, improving workplace characteristics, such as professional development opportunities, work organization, pace of work, and stress, plays a pivotal role in enhancing staff retention [36]. Our study confirmed this association, as the work environment in the healthcare facility significantly loaded onto the retention factor “Employment and career development”.

Professional development opportunities are critical determinants of health professionals’ decisions to migrate across Eastern Europe. Empirical studies indicate that medical specialists frequently seek enhanced career options, continuing education, and access to advanced medical knowledge beyond their home countries [29,33,40]. This trend was reflected in our analysis, with the professional advancement and work environment dimensions exhibiting strong factor loadings for items related to enhanced professional development opportunities. Migration decisions are often shaped by the availability of comprehensive and advanced training prospects [32].

The positive relationship between career-oriented migration and perceived enhancement in skill utilization highlights the interplay between professional aspirations and international mobility [40]. Conversely, career progression and workplace environment may encourage health professionals to stay in their home country. Our study demonstrated that satisfaction with achieved professional status and career prospects emerged as significant retention dimensions. A qualitative study among Hungarian health professionals highlights that professional commitment and organizational career pathways are fundamental to retention decisions [33].

Professional recognition constitutes a fundamental determinant of health workforce motivation, particularly in countries where professionals encounter status, autonomy, and compensation challenges [41]. Empirical evidence from Poland reveals that nurses, midwives, and physiotherapists have two primary concerns: low professional prestige and limited work autonomy [32]. These issues contribute to job dissatisfaction and may serve as push factors in the migration dynamics. Similar results were observed in the present study. Although the item “Improved recognition of the medical profession” had a modest contribution to the overall variance, it emerged as a significant migration driver. Likewise, enhanced professional recognition is a critical retention determinant of Lithuanian nurses [37]. The convergence of these findings underscores the dual role of professional recognition, serving as a migration driver and a retention mechanism.

The organization of the health system plays a pivotal role in facilitating the migration of health professionals. Outperforming health systems in destination countries serve as a strong pull factor, offering enhanced working conditions and career opportunities. In the present study, the advanced organization of the foreign health system constituted a significant motivator for Bulgarian health professionals. Boncea’s findings on Romanian healthcare workers have highlighted that the availability of modern equipment and advanced medical technologies enhances the attractiveness of destination countries [29]. The technological gap not only influences the quality of care but also shapes professional development and job satisfaction, making migration an appealing option for skilled professionals [42]. Workforce shortages in home countries also contribute to migration dynamics. Understaffed health systems contribute to increased workload and heightened stress among remaining professionals, potentially motivating them to consider employment abroad [33,43,44].

Our study identified family-related factors, notably improved partner career prospects and better educational opportunities for children, as significant determinants of migration intentions. Varga has also demonstrated how personal and social considerations affect migration decisions [45]. Additionally, professional networks abroad serve as an incentive, offering support through the integration of emigrants into new environments. This phenomenon is particularly evident in the case of Romanian physicians [46]. Furthermore, the prospect of improved quality of life is a compelling motivation for migration, encompassing not only better economic conditions but also improved prospects for family members and enhanced social stability [5,33].

However, some health professionals opt to remain in their home countries because of their families, social networks, and language barriers. These factors were particularly evident among nurses, as demonstrated by the present findings. The importance of family connections and personal dependencies in the country of origin has been further supported by research on health workers in the Southeast European Health Network Region [35]. Language barriers have also been identified as an important retention factor, particularly among experienced professionals [32].

The predominance of professional advancement and work environment as migration drivers suggests that policy interventions should prioritize improvements in working conditions and career development. Interventions targeting healthcare infrastructure, professional pathways, and organizational culture will benefit both physicians and nursing professionals. Socioeconomic and healthcare framework considerations accounted for the largest share of variance in the retention factor structure, whereas employment and career development aspects contributed less prominently. While financial motives were secondary migration drivers, they remained an important consideration for health professionals. Targeted strategies addressing financial incentives and workplace safety may prove particularly effective for retaining nursing professionals, as personal considerations and social environment exerted a slightly stronger influence on their decision to remain in Bulgaria. Addressing the driving and retention factors can help mitigate the brain drain and enhance workforce sustainability. Such measures strengthen the capacity of the health system to deliver equitable and efficient health services, thereby improving health outcomes for the population.

The primary methodological contribution of this study lies in the systematic identification of latent constructs underlying migration and retention factors, rather than treating individual predictors as independent variables. While previous studies [47,48] have documented various migration drivers and retention determinants in Bulgaria, they have not empirically validated the theoretical dimensionality of these factors. By demonstrating how multiple drivers coalesce into distinct constructs, this study offers a more parsimonious framework for understanding health professional migration.

The present study has several limitations that should be considered when interpreting the findings. First, the cross-sectional design provided only a “snapshot” of health professionals’ attitudes and motivations at a specific time point. This design precludes any assessment of how these views evolve in response to policy changes. Second, the study was conducted during the COVID-19 pandemic, which may have influenced respondents’ perspectives, shifting their focus toward infection control, burnout mitigation, or emergency preparedness. Third, the use of an online survey format, while increasing the response rate, may have introduced selection bias: participants with stronger opinions or more flexible schedules were potentially overrepresented. Finally, the study focused exclusively on four health professional categories—physicians, dentists, nurses, and midwives. Although these groups play central roles in health service delivery, they do not encompass the full spectrum of healthcare workers. Expanding the professional sample in subsequent studies would enhance generalizability and provide a more comprehensive view of health professional migration.

## 5. Conclusions

Our study identified the underlying structure of migration intentions and retention factors among Bulgarian health professionals through EFA. Three dimensions of migration intentions emerged: professional advancement and work environment; financial incentives; and family benefits. Professional development and working conditions constituted the primary driver of emigration intentions, while economic and family-related factors played secondary roles. The retention structure similarly comprised three dimensions: socioeconomic and healthcare framework; employment and career development; and personal considerations and social environment. The dominant influence of national-level conditions and health system organization indicates that macro-level determinants constitute the primary basis for retention decisions.

Comparative analyses revealed no significant differences between physicians and nursing professionals regarding professional, systemic, or workplace-related factors. Nursing professionals attributed greater importance to financial incentives in migration decisions and to personal and social environment considerations in retention decisions. While structural and professional conditions influence both groups similarly, nurses demonstrate heightened sensitivity to remuneration and social workplace aspects.

The findings underscore the importance of addressing both driving and enhancing retention factors to mitigate the outflow of the health workforce. Consistent monitoring of migration intentions can inform the development of targeted, evidence-based retention strategies. Future research should examine the stability of these factor structures across health facility types and regional contexts within Bulgaria.

## Figures and Tables

**Table 1:** Sample characteristics (N = 338).

Variable	Frequency (n)	Proportion (%)
**Gender**		
Male	73	21.6
Female	265	78.4
**Age** (years)		
22–35	63	18.6
36–45	69	20.4
46–55	111	32.8
56–65	72	21.3
65+	23	6.8
**Profession**		
Physician	158	46.8
Dentist	33	9.8
Nurse	131	38.8
Midwife	16	4.7
**Work experience** (years)		
<3	33	9.8
3–10	47	13.9
11–20	83	24.6
>20	175	51.8
**Marital status**		
Married	191	56.5
Single	56	16.6
Civil partnership or cohabitation	60	17.8
Divorced or widowed	31	9.2
**Children**		
Under 18 years old	109	32.2
Above 18 years old	154	45.6
Without children	75	22.2
**Place of residence**		
Capital	97	28.7
District city	167	49.4
Small town	61	18.0
Village	13	3.8

**Table 2:** Factor structure of migration drivers.

Variables	Factor Loadings		
	Factor 1: Professional Advancement and Work Environment	Factor 2: Financial Incentives	Factor 3: Family Benefits
Access to advanced technologies	**0.930**	−0.114	−0.023
Favorable working conditions	**0.921**	−0.019	−0.020
Superior prospects for training and specialization	**0.807**	0.076	−0.084
Advanced health system organization	**0.807**	−0.106	0.049
Enhanced opportunities for professional development	**0.763**	0.203	−0.059
Upgraded economic environment	**0.656**	0.087	0.099
Improved recognition of the medical profession	**0.650**	−0.021	0.128
Higher remuneration	−0.059	**0.986**	0.011
Higher standard of living	0.030	**0.918**	−0.018
Educational prospects for children	−0.027	0.003	**0.910**
Improved prospects for the partner	0.042	−0.023	**0.886**
Financial support for the family (remittances)	0.280	0.186	0.274

Note: Exploratory factor analysis rotated pattern matrix. All significant loadings in bold.

**Table 3:** Retention factor structure.

Variables	Factor Loadings		
	Factor 1: Socioeconomic and Healthcare Framework	Factor 2: Employment and Career Development	Factor 3: Personal Considerations and Social Environment
Health infrastructure development	**0.972**	0.018	−0.143
Health system strengthening	**0.847**	0.148	−0.172
Partner’s career path	**0.557**	0.066	0.160
National economic prospects	**0.553**	0.087	−0.022
Quality of life in Bulgaria	**0.519**	0.090	0.275
Work environment in the healthcare facility	−0.053	**0.863**	0.088
Prospects for career progression	0.140	**0.755**	−0.031
Earnings received	0.160	**0.706**	−0.010
Achieved professional status	0.143	**0.547**	0.062
Family and social networks	−0.283	0.165	**0.564**
Safety and security in Bulgaria (absence of military conflicts, internal unrest)	0.202	−0.004	**0.498**
Language barrier	0.020	−0.038	**0.404**
Local schooling opportunities	0.394	−0.104	0.491

Note: Exploratory factor analysis rotated pattern matrix. All significant loadings in bold.

## Data Availability

The data presented in this study are available on reasonable request from the corresponding author due to ethical restrictions.

## Abbreviations

The following abbreviations are used in this manuscript:

EFA	Exploratory Factor Analysis
EU	European Union
WHO	World Health Organization
OECD	Organization for Economic Co-operation and Development
MOHPROF	Mobility of Health Professionals project
KMO	Kaiser–Meyer–Olkin
